# Consequence of insertion trauma – effect on early measurements when using intracerebral devices

**DOI:** 10.1038/s41598-019-47052-4

**Published:** 2019-07-23

**Authors:** Ted Carl Kejlberg Andelius, Mette Vestergård Pedersen, Nikolaj Bøgh, Camilla Omann, Vibeke Elisabeth Hjortdal, Michael Pedersen, Kasper Jacobsen Kyng, Tine Brink Henriksen

**Affiliations:** 1 Department of Paediatrics, Aarhus University Hospital, Palle Juul-Jensens Blvd. 99, 8200 Aarhus N, Denmark; 2 Department of Cardiothoracic and Vascular Surgery, Aarhus University Hospital, Palle Juul-Jensens Blvd. 99, 8200 Aarhus N, Denmark; 3 Department of Comparative Medicine, Aarhus University Hospital, Palle Juul-Jensens Blvd. 99, 8200 Aarhus N, Denmark

**Keywords:** Brain injuries, Sensors and probes

## Abstract

There are a variety of devices that quantify biological properties of cerebral tissue. Installing such device will cause a local insertion trauma, which will affect early measurements. Current literature proposes minimum one hour of observation before acquiring first measurements when using microdialysis. It is unknown whether this applies to other intracerebral devices. We therefore aimed to investigate time needed to reach steady state when using microdialysis and two intracerebral probes in a piglet model. Ten newborn piglets less than 24 hours of age were anaesthetized. Two probes (Codman and OxyLite/OxyFlo) and a microdialysis catheter (CMA Microdialysis) were installed 10 mm into the left hemisphere. Probes measured intracranial pressure, cerebral blood flow, and oxygen tension. The microdialysis catheter measured lactate, glucose, glycerol, and pyruvate. Measurements were acquired hourly for 20 hours. Lactate and glycerol peaked immediately after insertion and reached steady state after approximately four hours. Glucose, pyruvate, cerebral blood flow, and intracranial pressure reached steady state immediately. Oxygen tension reached steady state after 12 hours. With time, interindividual variability decreased for the majority of measurements. Consequently, time to stabilization after insertion depends on the choice of device and is crucial to obtain valid baseline values with high degree of precision.

## Introduction

To quantify biological properties of cerebral tissue, microdialysis and intracerebral probes are used^1^. In paediatric research, these devices are used in experimental animal studies to investigate the intracerebral consequence of perinatal hypoxia and ischemia and specific treatments^2,3^. In neurocritical care, microdialysis and intracerebral probes are used to detect secondary ischemia in patients with intracerebral bleeding, or to measure the influence of different interventions in animal experiments^4,5^. Accordingly, information on metabolism, intracranial pressure (ICP), cerebral blood flow (CBF), and oxygenation are essential for cerebral monitoring in both the clinical and experimental setting^6^. To map metabolism, microdialysis can be used with a standard panel of glucose, lactate, glycerol, glutamate, and pyruvate^7^. ICP can be measured invasively by a strain-gauge device^8^. Cerebral oxygenation and CBF can be measured directly through a probe inserted into the brain parenchyma^3^. However, when installing a microdialysis catheter or a probe, a local insertion trauma will occur which will affect early measures.

When using microdialysis in human muscle tissue, a minimum of two hours of observation was required for values to stabilize^9,10^. Sørensen *et al*. found a reduction in lactate, glycerol, and glutamate when comparing microdialysis measurements acquired at 20 minutes with those obtained at 40 minutes after insertion in muscle tissue^11^. Even longer time to stabilization have been reported when measures are carried out in cerebral tissue^12,13^. Mellergård *et al*. investigated the impact of microdialysis-catheter placement in patients with severe brain injury^12^. They found stable values of glycerol, lactate and pyruvate immediately after insertion; but substantial changes in glutamate and several cytokines and chemokines up to 48 hours after insertion^12^. *Consensus statement from the 2014 International Microdialysis Forum* states that during the *first* hour, microdialysis measurements should not be used due to interference from the insertion trauma^7^. There is a lack of data on the consequence of insertion trauma for other devices used in cerebral tissue. The aim of this newborn-piglet study was to determine whether one hour of observation after insertion of two intracerebral probes, and a microdialysis catheter was sufficient to reach steady state.

## Results

All 10 piglets survived for the 20-hour observation period. One piglet had a malfunctioning microdialysis catheter and data was not obtained. ICP measurements were not recorded in two piglets due to probe failure. Vital signs and blood-gas values remained stable though the observation period (Table 1).

**Table 1:** Vital signs and blood-gas values for 10 piglets during the 20-hour observation period presented as mean values with range.

	1^st^ hour	6^th^ hour	12^th^ hour	18^th^ hour
**Vital signs:**				
Heart rate (min^−1^)	139 (98–176)	168 (130–230)	164 (130–250)	158 (126–231)
Mean arterial pressure (mmHg)	46.2 (34.0–60.0)	41.8 (39.0–46.0)	39.9 (34.0–46.0)	42.0 (35.5–47.0)
Rectal temperature (°C)	38.0 (36.4–39.3)	39.0 (38.3–39.7)	38.9 (38.2–39.3)	38.8 (38.2–39.4)
**Blood-gas values:**				
pH	7.6 (7.4–7.7)	7.6 (7.5–7.6)	7.5 (7.4–7.6)	7.6 (7.5–7.6)
pCO_2_ (kPa)	4.1 (2.7–6.0)	4.0 (3.3–4.7)	4.3 (3.6–5.0)	3.9 (3.3–4.4)
pO_2_ (kPa)	10.8 (8.1–14.7)	11.3 (7.9–19.2)	10.4 (8.4–12.6)	12.0 (9.1–17.6)
Lactate (mmol/L)	2.1 (1.3–3.5)	1.4 (1.1–1.9)	1.3 (1.0–1.6)	1.5 (1.1–1.8)
Glucose (mmol/L)	7.4 (4.1–11.8)	5.3 (4.5–6.3)	6.4 (4.0–13.3)	5.8 (4.3–7.6)
Na^2+^ (mmol/L)	137.1 (135.0–140.0)	135.3 (132.0–141.0)	131.5 (123.0–141.0)	128.0 (124.0–134.0)
K^+^ (mmol/l)	3.5 (2.4–4.3)	3.8 (2.8–4.5)	4.0 (3.0–5.7)	3.8 (2.8–4.1)

Blood-gasses were sampled 1, 6, 12, and 18 hours (±1 hour) after insertion.

### Microdialysis

**Figure 1:**
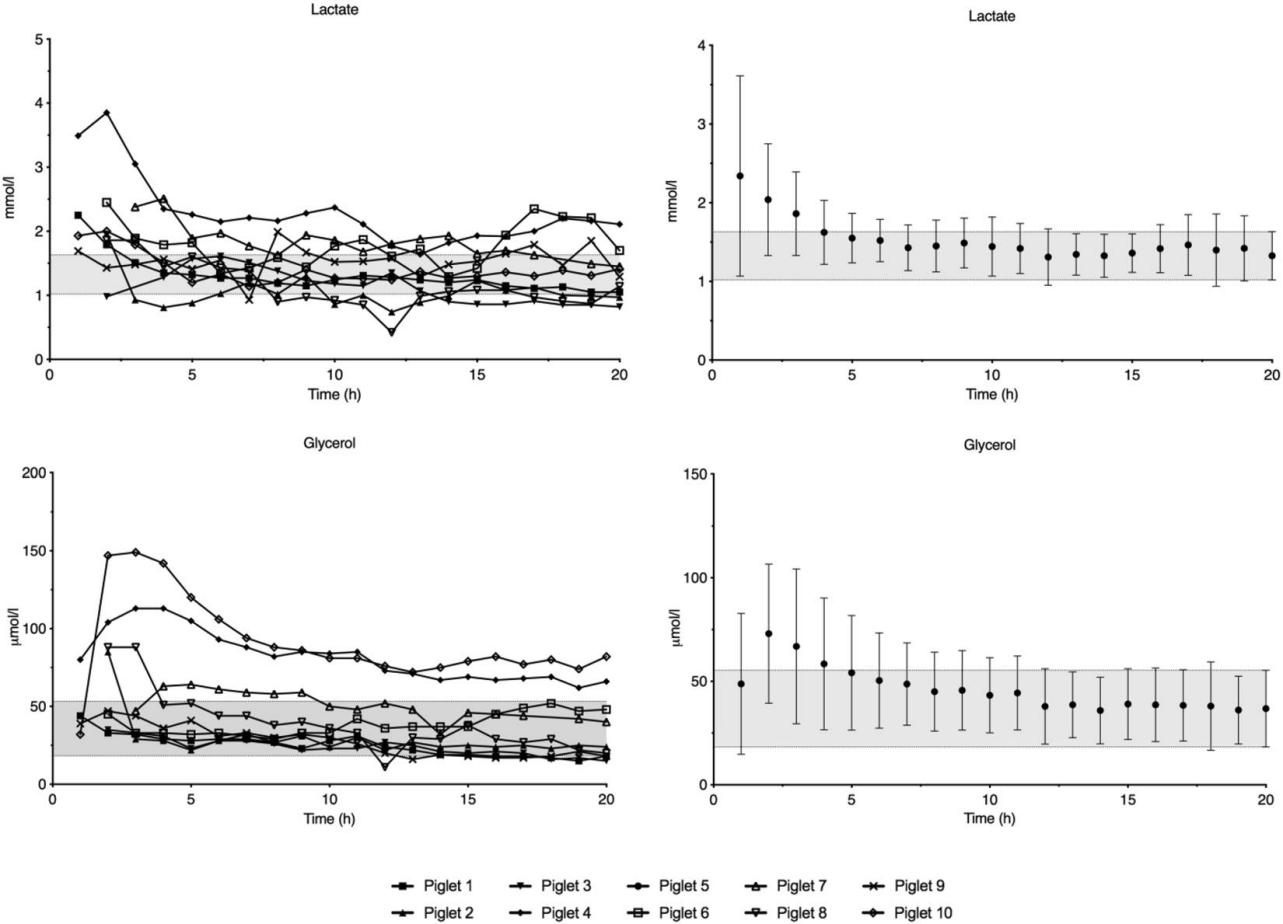
Average lactate and glycerol concentrations in 9 piglets during the 20-hour observation period. Data are means with 95% confidence interval. The 95% confidence interval of the measurement at 20 hours is marked with a grey field.

**Figure 2:**
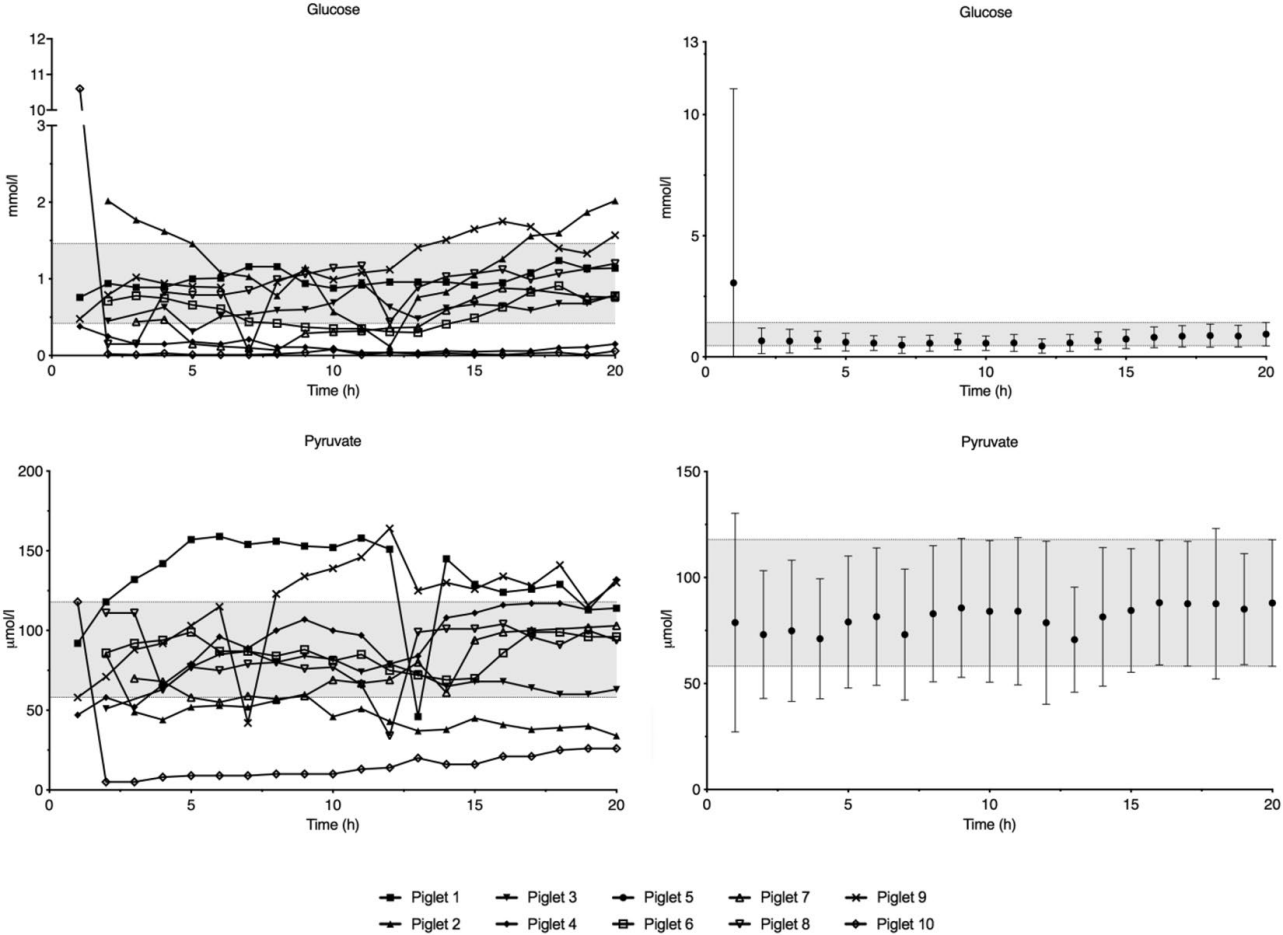
Average glucose and pyruvate concentrations in 9 piglets during the 20-hour observation period. Data are means with 95% confidence interval. The 95% confidence interval of the measurement at 20 hours is marked with a grey field.

Lactate concentration peaked one hour after insertion and decreased to reach steady state at four hours (Fig. 1). Glycerol concentration increased to a peak at two hours and reached steady state after five hours of observation (Fig. 1). Except for one animal with an initially very high intracerebral glucose concentration, steady state was reached after one hour of observation for both glucose and pyruvate (Fig. 2). The interindividual variation of lactate and glycerol decreased during the first four and seven hours, respectively, while glucose and pyruvate remained constant throughout the observation period (Supplementary Data S1).

### Probes

**Figure 3:**
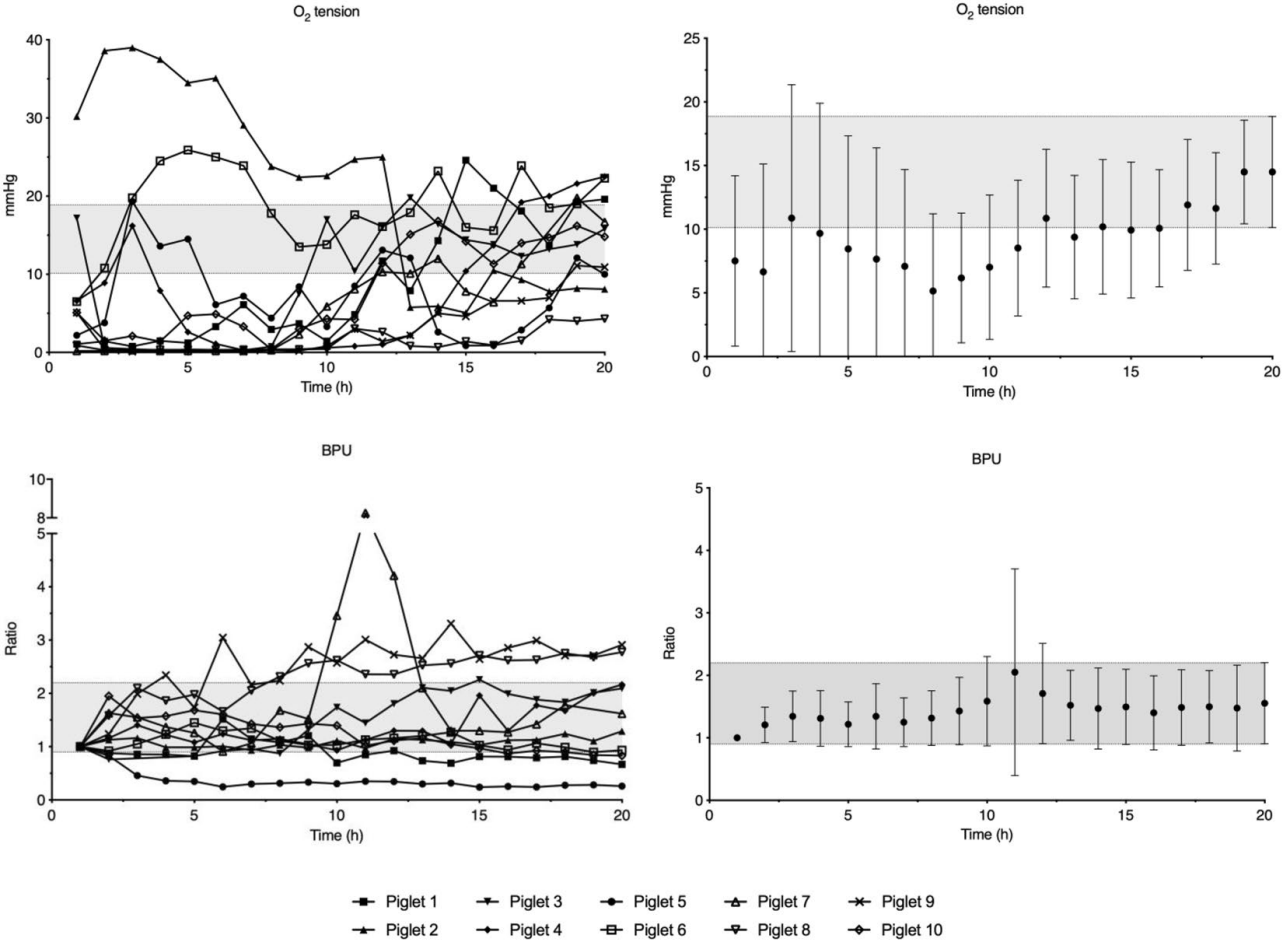
Average measurements of O_2_ tension and cerebral blood flow in 10 piglets during the 20-hour observation period. Data are means with 95% confidence interval. The 95% confidence interval of the measurement at 20 hours is marked with a grey field. BPU; blood perfusion units.

**Figure 4:**
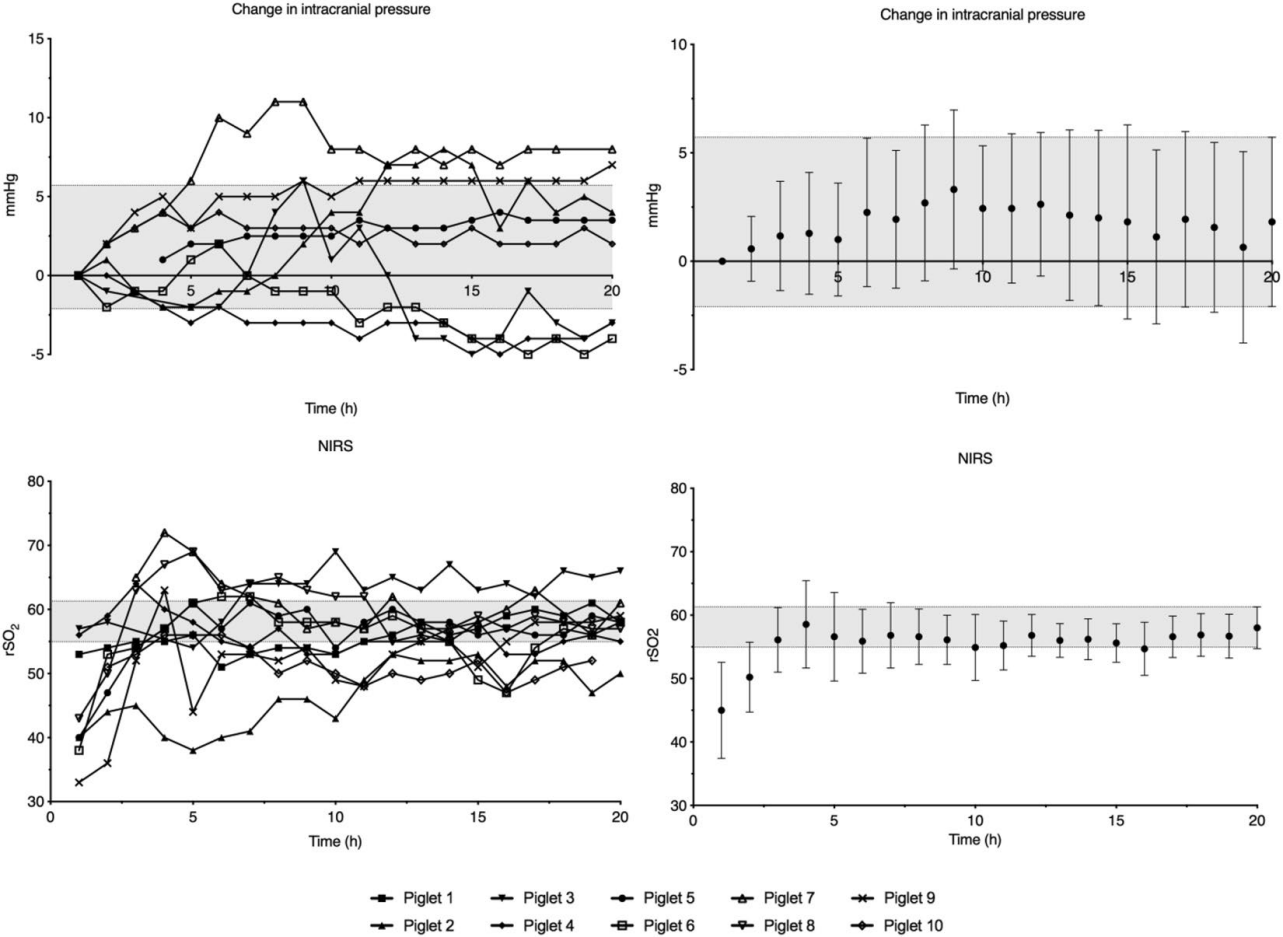
Average measurements of change in intracranial pressure for 8 piglets and NIRS for 10 piglets during the 20-hour observation period. Data are means with 95% confidence interval. The 95% confidence interval of the measurement at 20 hours is marked with a grey field. NIRS; near infrared spectroscopy.

Mean oxygen tension increased through the whole observation period despite normal blood-gas values, although steady state was reached after 12 hours (Fig. 3). Some animals required 10–15 hours of observation before stable oxygen-tension values could be detected (Fig. 3). Interindividual variation of oxygen tension decreased until 10 hours of observation (Supplementary Data S1). Change in CBF and ICP reached steady state in the 2^nd^ hour (Figs 3 and 4). After insertion of the probes, animals presented with a minor change in ICP and CBF, thus resulting in an increased interindividual variation for both measures (Supplementary Data S1). After approximately 10 hours of observation, ICP stabilized as presented in the raw data of Fig. 4.

### Near Infrared Spectroscopy (NIRS)

NIRS values increased and researched steady state after three hours of observation (Fig. 4). Interindividual variation decreased during the first 11 hours of observation (Supplementary Data S1).

## Discussion

We showed that sufficient observation time to reach steady state is essential to acquire valid baseline measurements when using intracerebral probes and microdialysis. Time needed to reach steady state was longer than expected for the majority of measurements and varied depending on the device and the specific metabolite measured. We also showed that adequate observation time is important to get homologous baseline values, as interindividual variability decreased for the majority of measures during the first hours of observation.

Benveniste *et al*. showed that the recovery from a microdialysis catheter will initially be increased after insertion, thus potentially producing falsely increased values during the first 30–60 minutes^14^. They also showed that the insertion trauma will cause an acute leak over the blood-brain barrier during the first 10 minutes after insertion, which will affect early measures further^14^. These acute changes are consistent with our results. Further, microdialysis recovery will be affected by other factors related to the technical details (e.g., membrane length and flow rate)^7^. A flow rate of 0.3 μL/min is commonly used^7^. To ensure sufficient sample volume, we chose a flow rate of 0.5 μL/min. This needs to be acknowledged in study-to-study comparison of absolute values, but should not affect overall trend.

Glycerol is considered a marker of phosolipid degeneration and cell wall destruction^4^. We found a delay with glycerol concentration peak two hours after the insertion trauma followed by a *decrease* towards steady-state values. A similar pattern was seen with regards to lactate (Fig. 1). Lactate production may be a consequence of mitochondrial dysfunction, and lactate concentrations were increased shortly after insertion and then *decreased* to reach steady state^15^. The insertion trauma thus appeared to cause cell lysis and metabolic compromise with release of metabolites, which may have influenced early microdialysis measures. O_2_ tension *increased* during our observation period and required the longest time to reach steady state of all measurements (Fig. 3). O’Hara *et al*., used a similar device, and reported an average of 52 minutes of observation before acquiring stable values^16^. They found haemorrhage in the insertion canal of the probe on post-mortem examination, which together with tissue compression, temperature changes, and location of the probe may have affected early readings^16^. They retracted the probe 0.5 mm after insertion to relieve pressure from the tip; a procedure we did not perform. We placed the piglet in a supine position after placement of the probe, which may have resulted in additional tissue compression in our study. We also found blood and microscopic bone fragments inside the probe canal on post-mortem examination and we believe that this may be a likely cause of the prolonged time to steady state seen in our animals. Compared to glycerol and lactate concentration, the insertion trauma may have caused *low* readings right after insertion due to bleeding and potential blockage of the tip of the device – rather than producing metabolites picked up by the device resulting in early *elevated* readings. These findings depict two different mechanisms on how the insertion trauma may interfere with early readings.

Cerebral oximetry by NIRS is known to have a wide intraindividual variability and may therefore best be used as a trend monitor^17^. Surprisingly, NIRS measurements increased for all animals during the first hours of observation and reach steady state at 3 hours (Fig. 4). Placed on the contralateral side of the head, NIRS measures should not be directly affected by the insertion of the probes or microdialysis catheter. Mechanical damage caused by the insertion of a device into cerebral tissue will trigger *spreading depressions*, i.e., short depolarizations with neuronal swelling, ion flux, change in CBF, increased glucose consumption, and increased oxygen demand^18^. Most of the harmful effects of spreading depressions resolve within minutes after the insertion trauma^19^. However, oxygen demand may be increased for up to two hours after recovery^20^. This is in line with our findings as spreading depressions may alter oxygen demand on the contralateral side due to their migrating nature, and proposes the possibility that inserting a device into cerebral tissue might also affect early reading from extracerebral devices such as NIRS^20^.

We installed the devices into healthy cerebral tissue. In neurocritical care, microdialysis is often installed during operation for various pathologies (e.g., traumatic brain injury or sub arachnoid haemorrhage)^21^. When used in perilesional tissue compared to healthy tissue in patients with traumatic brain injury, microdialysis measurements showed altered baseline values and a different reaction to change in other physiological parameters such as intracranial pressure and oxygenation^22^. Although device size, shape, and stiffness may influence the severity of the insertion trauma, little is known on whether the number of devices placed in close proximity might influence the mearuements^23^. To ensure sampling of the same anatomical location, we used an inter-device distance of 3 mm. Thus, the insertion trauma from one device might have influenced the tissue surrounding the neighbouring device.

Researchers are ethically obliged to utilize as few animals as possible, according to the “reduction” in the 3R-principple, as originally described by Russell and Burch in 1959^24^. In this study, we show that sufficient observation time before interventions will result in a decreased interindividual variation and thus fewer animals needed to obtain the same power when comparing two or more groups (Supplementary Data S1).

The insertion trauma is most likely affected by the skill and dexterity of the researcher placing the device. This might be limited by the use of a stereotactic frame, which was not used in the current study. Another weakness is related to the precision of the device placement. We aimed to standardize device placement through measurements made before hand on autopsy in pilot animals. The final location was only verified in a few animals by macroscopic examination post-mortem. To reduce variation, all devices were placed by the same researcher (TCKA) according to standardized measurements made beforehand. A strength of this study is the long observation time. This allowed for repeated measurements, which for some animals were needed to get the stabile baseline data with little inter-individual variation. Our findings are likely generalizable to the use of intracerebral devices in other animal species or humans. Steady state was determined by means of 95% confidence intervals after 20 hours observation, as we expected the impact of the insertion trauma to be minimal at this time. This simple strategy of determining steady state, combined with graphing of raw data along with the presentation of averaged data, was chosen to allow for critical revision of the temporal changes by others.

Accordingly, one hour of observation after insertion was insufficient to acquire stable measurements in our setup. The observation time depends on the choice of device and the specific metabolite measured. The early measurements will not only be affected by the insertion trauma but also by other factors, such as the state of the cerebral tissue, recovery of microdialysis, type and number of devices. This study underlines the importance of appropriate observation time after insertion when using intracerebral devices. We demonstrate that appropriate observation time is crucial to retrieve information with a minimum of interindividual variation, which in turn will reduce the number of animals needed to detect an effect of a given intervention.

## Methods

We used a neonatal piglet model, previously described by our group^25^. The project was conducted according to the guidelines given by the Danish Animal Experimentation Inspectorate and was approved by this institution (2016–15–0201–01146). The study comprised 10 newborn Danish landrace piglets, 12–24 hours old, with a mean body weight of 1.7 (1.4–2.0) kg and mean haemoglobin of 5.0 (4.3–6.8) mmol/L. The project is reported in accordance with the ARRIVE guidelines^26^.

### Anaesthesia and monitoring

The piglets were anesthetized by inhalation of 2–4% sevoflurane. Intravenous access was acquired through an ear vein and a bolus of propofol 10 mg/kg, fentanyl 30 μg/kg, and rocuronium 1 mg/kg was administered. The animals were intubated ventilated with a target end-tidal CO_2_ (ECO_2_) of 4.5–5.5 kPa. After intubation, sedation and analgesia was maintained by continuous infusion of propofol 4–12 mg/kg/h and fentanyl 10–20 μg/kg/h. For monitoring purposes, blood sampling, and fluid and glucose administration, umbilical venous and arterial catheters were placed. Electrolyte and glucose levels were kept within the normal range by infusion with 0.72% NaCl (5 mmol/l K and 10% glucose) at 5–10 ml/kg/h. Mean arterial blood pressure (MAP), heart rate, oxygen saturation (SatO_2_%), fraction of inspired oxygen (FiO_2_), ECO_2_, and temperature were monitored and transferred to a computer by dedicated software (Datex Ohmeda S/5 Collect, Finland). Core temperature was measured by a rectal thermometer. All animals were treated with subcutaneous benzylpenicillin 15,000 IE/kg once every 12 hours. Plasma electrolytes, glucose, and blood-gas values was regularly measured during the 20-hour observation period and fluids, electrolytes, and ventilator settings was adjusted accordingly. We aimed to maintain a target MAP above 40 mmHg. Mean blood pressures below the target MAP were treated by first reducing anaesthesia and analgesia infusion. If hypotension persisted the animal was treated with noradrenaline infusion 0.25–1.5 μg/kg/min^27^.

### Intracerebral measurements

Using a cannula (diameter of 2.1 mm for the first and 1.5 mm for the second and third hole) three holes of 10 mm depth where made through the scalp. The most rostral hole was made 5 mm posterior to a straight line between the eyes and 10 mm laterally of the midline. The two additional holes were made 3 mm apart, the first being 3 mm posterior to the first hole. All three holes were made on the left side of the midline, targeting the left parasagittal cortex. In the most rostral hole a probe measuring ICP was installed (Codman, Sweden). The ICP probe was calibrated to zero in room air before insertion. A probe measuring CBF by laser-doppler technique (presented as blood perfusion units [BPU]), temperature, and oxygen tension was installed in the second hole (OxyLite/OxyFlo, Oxford Optronics, UK). In the third hole, a CMA 20 Elite microdialysis catheter (4 mm membrane length) with central nervous system, sterile isotonic perfusion fluid with a flowrate 0.5 μl/min, was installed and connected to a CMA 402 syringe pump (CMA Microdialysis, Kista, Sweden). The microdialysate was analysed for lactate, pyruvate, glucose, and glycerol on a CMA 600 microdialysis analyser. The probes and the microdialysis catheter were then secured to the scalp. To serve as a non-invasive cerebral measurement, a near infrared spectroscopy (NIRS) neonatal somasensor (Medtronic, MN, USA) was placed on the parietal region on the right side of the scalp. The piglets were then put in a supine position.

### Experimental protocol

After placement of the intracerebral probes and microdialysis catheter, animals were allowed to rest for 30–60 minutes. The first measurements were then acquired (defined as time point “1”). Then animals were observed for 20 hours. Measurements were acquired once every hour. At the end of the experiment, the animals were euthanized through a lethal injection with pentobarbital 400 mg/ml, 0.4 ml/kg.

### Statistics

From the Oxford-Optronics probe, measurements were registered for 5–10 minutes once every hour and a mean value during that time was noted as a value for that hour. Data from the ICP probe are presented as change in mmHg from insertion. When calculating change in ICP and CBF (BPU), measurements from the 1^st^ hour were used as the reference. Due to the exploratory nature of this study, we applied the *Resource Equation Method* to determine sample size^28^. Despite a small sample size and risk of loss of animals throughout the study, we estimated that 10 animals was sufficient to complete the aim of this study. We expected that all three devices would have reached baseline at 20 hours of observation. Therefore, the mean with 95% confidence intervals (CI) for that time point were used as a reference when determining at what time point steady state was reached. All intracerebral measurements are presented as mean with 95% CI as well as raw data for each of the measures. 95% CI at 20 hours are marked with a grey field in each graph. Demographic data are presented as mean with range. Within-population variations are presented as standard deviations in Supplementary Data S1.

## Supplementary information

Supplementary data S1

## Data Availability

The datasets generated and analysed during the current study are available from the corresponding author on request.
